# Improved Convolutive and Under-Determined Blind Audio Source Separation with MRF Smoothing

**DOI:** 10.1007/s12559-012-9185-9

**Published:** 2012-09-07

**Authors:** Rafał Zdunek

**Affiliations:** Institute of Telecommunications, Teleinformatics, and Acoustics, Wroclaw University of Technology, Wybrzeze Wyspianskiego 27, 50-370 Wroclaw, Poland

**Keywords:** Blind source separation, Nonnegative matrix factorization, Expectation-maximization, Markov random field, Simultaneous auto-regression

## Abstract

Convolutive and under-determined blind audio source separation from noisy recordings is a challenging problem. Several computational strategies have been proposed to address this problem. This study is concerned with several modifications to the expectation-minimization-based algorithm, which iteratively estimates the mixing and source parameters. This strategy assumes that any entry in each source spectrogram is modeled using superimposed Gaussian components, which are mutually and individually independent across frequency and time bins. In our approach, we resolve this issue by considering a locally smooth temporal and frequency structure in the power source spectrograms. Local smoothness is enforced by incorporating a Gibbs prior in the complete data likelihood function, which models the interactions between neighboring spectrogram bins using a Markov random field. Simulations using audio files derived from stereo audio source separation evaluation campaign 2008 demonstrate high efficiency with the proposed improvement.

## Introduction

Blind source separation (BSS) aims to recover unknown source signals from observed mixtures with or without very limited information about their mixing process. BSS problems have been addressed in many previous studies, for example, [1–12], which were motivated by several real-world applications.

In a cocktail-party problem, microphones receive noisy mixtures of acoustic signals that propagate along multiple paths from their sources. In a real scenario, the number of audio sources may be greater than the number of microphones, audio sources may have different timbres and similar pitches, and audio signals may be only locally stationary.

A convolutive and under-determined mixing approach needs to be adopted to model this problem. There are several techniques for solving convolutive unmixing problems [13]. Some of these [14] operate in the time-domain by solving the alternative finite impulse response (FIR) inverse model using independent component analysis (ICA) methods [2]. Another method is to extract meaningful features from the time-frequency (TF) representations of mixtures. This approach seems to be more efficient than the ICA-based techniques especially when the number of microphones is lower than the number of sources. Acoustic signals are usually sparse in the TF domain, so the source signals can be separated efficiently even if they are partially overlapped and the problem is under-determined. These features can be extracted using several techniques, including TF masking [15, 16], frequency bin-wise clustering with permutation alignment (FBWC-PA) [17, 18], subspace projection [19], hidden Markov models (HMM) [20], interaural phase difference (IPD) [21], nonnegative matrix factorization (NMF) [22, 23], and nonnegative tensor factorization (NTF) [24].

Nonnegative matrix factorization [25] is a feature extraction method with many real-world applications [26]. A convolutive NMF-based unmixing model was proposed by Smaragdis [22]. Ozerov and Fevotte [23] developed the EM-NMF algorithm, which is suitable for unsupervised convolutive and possibly under-determined unmixing of audio sources using only stereo observations. Their model of the sources was based on the generalized Wiener filtering model [27–29], which assumes that each source is locally stationary and that it can be expressed in terms of superimposed amplitude-modulated Gaussian components. Thus, a power spectrogram of each source can be factorized into lower-rank nonnegative matrices, which facilitates the use of NMF for estimating the frequency and temporal profiles of each latent source component. In the TF representation, the latent components are mutually and individually independent across frequency and time bins. However, this assumption is very weak for any adjacent bins because real audio signals have locally smooth frequency and temporal structures.

Motivated by several papers on smoothness [26, 28, 30, 31] in BSS models, we attempt to further improve the EM-NMF algorithm by enforcing local smoothness both in the frequency and temporal profiles of the NMF factors. Similar to [28, 30, 32], we introduce a priori knowledge to the NMF-based model using a Bayesian framework, although our approach is based on a Gibbs prior with a Markov random field (MRF) model to describe pairwise interactions among adjacent bins in spectrograms. As demonstrated in [33], the MRF model with Green’s function, which is well known in many tomographic image reconstruction applications [34], can improve the EM-NMF algorithm. In this paper, we extend the results presented in [33] using other smoothing functions, particularly a more flexible simultaneous autoregressive (SAR) model that is more appropriate in term of hyperparameter estimation and computational complexity.

The rest of this paper is organized as follows. The next section reviews the underlying separation model. Section 3 is concerned with MRF smoothing. The optimization algorithm is described in Sect. 4. Audio source separation experiments are presented in Sect. 5. Finally, the conclusions are provided in Sect. 6.

## Model

Let *I* microphones receive signals that can be modeled as a noisy convolutive mixture of *J* audio signals. The signal received by the *i*-th microphone ($i=1, \ldots, I$) can be expressed as

1 $$ \tilde x_i(t)=\sum_{j=1}^J \sum_{l=0}^{L-1} \tilde a_{ijl} \tilde s_j(t - l)+\tilde n_i(t), $$

where $\tilde a_{ijl}$ represents the corresponding mixing filter coefficient, $\tilde s_j(t)$ is the *j*-th source signal ($j=1, \ldots, J$), $\tilde n_i(t)$ is the additive noise, and *L* is the length of the mixing filter.

In the TF domain, the model (1) can be expressed as

2 $$ x_{ift}=\sum_{j=1}^J a_{ijf} s_{jft}+n_{ift}, \quad \hbox{or equivalently}, \quad \varvec{X}_f=\varvec{A}_f \varvec{S}_f +\varvec{N}_f, $$

where ${\varvec{X}_f=[x_{ift}]_f \in {\mathbb C}^{I \times T}, \varvec{A}_f=[a_{ijf}]_f \in {\mathbb C}^{I \times J}, \varvec{S}_f=[s_{jft}]_f \in {\mathbb C}^{J \times T}, \varvec{N}_f=[n_{ift}]_f \in {\mathbb C}^{I \times T}}$, and $f=1, \ldots, F$ is the index of a frequency bin.

The noise *n*_*ift*_ is assumed to be stationary and spatially uncorrelated, i.e,

3 $$ n_{ift} \sim {\mathcal{N}}_c(0, \varvec{\Upsigma}_n), $$

where $\varvec{\Upsigma}_n=\hbox{diag}\left ([\sigma^2_{i}] \right)$ and $\mathcal{N}_c(0, \varvec{\Upsigma}_n)$ is a proper complex Gaussian distribution with a zero-mean and the covariance matrix $\varvec{\Upsigma}_n$.

Benaroya et al. [27] described an audio source $\tilde{s}(t)$ as a superimposed amplitude-modulated Gaussian process:

4 $$ \tilde{s}(t)=\sum_{r=1}^R h_r(t) \tilde{w}_r(t), $$

where *h*_*r*_(*t*) is a slowly varying amplitude parameter in the *r*-th component ($r=1, \ldots, R$), and $\tilde{w}_r(t)$ is a stationary zero-mean Gaussian process with the power spectral density σ_*r*_^2^ (*f*). The TF representation of (4) leads to

5 $$ s(f,t) \sim {\mathcal{N}}_c \left (0, \sum_{r=1}^R h_r(t) \sigma^2_{r}(f) \right). $$

The power spectrogram of (5) is given by |*s*_*ft*_|^2^ = ∑_*r*=1_^*R*^ *w*_*fr*_ *h*_*rt*_, where *w*_*fr*_ = σ_*r*_^2^(*f*). Thus, the spectrogram of the *j*-th source $\tilde{s}_j(t)$ can be factorized as follows:

6 $$ |\varvec{S}_j|^2=\varvec{W}_j \varvec{H}_j, $$

where ${\varvec{S}_j \in {\mathbb C}^{F \times T}, \varvec{W}_j \in {\mathbb R}_+^{F \times R_j}, \varvec{H}_j \in {\mathbb R}_+^{R_j \times T}, R_j}$ is the number of latent components in the *j*-th source, and ${\mathbb R}_+$ is the nonnegative orthant of the Euclidean space. The column vectors of $\varvec{W}_j$ represent the frequency profiles of the *j*-th source, while the row vectors of $\varvec{H}_j$ are the temporal profiles.

Févotte et al. [28] transformed the model (5) to the following form:

7 $$ s(f,t)=\sum_{r=1}^R c_r (f,t) $$

where $$c_r(f,t) \sim \mathcal{N}_c \left (0, h_r(t) \sigma^2_{r}(f) \right)=\mathcal{N}_c \left (0, \varvec{\Upsigma}_{ft}^{(c)} \right)$$. Thus,

8 $$ \varvec{\Upsigma}_{ft}^{(c)}=\hbox{diag}\left ([w_{fr} h_{rt}]_r \right)=\hbox{diag}\left (\left [|c_{rft}|^2 \right]_r \right), $$

and $\left [\sum\nolimits_{r=1}^{R} |c_{rft}|^2 \right]=\varvec{W}\varvec{H}$, where ${\varvec{W} \in {\mathbb R}^{F \times R}_+, \varvec{H} \in {\mathbb R}^{R \times T}_+}$. Consequently, the model (2) can be expressed as

9 $$ x_{ift}=\sum_{r=1}^{R} \bar{a}_{irf} c_{rft}+n_{ift}, \quad\hbox{ where} \quad s_{jft}=\sum_{r \in {\mathfrak{R}}_j} c_{rft}, $$

${R=|\mathfrak{R}|}$ is the number of entries in the set ${{\mathfrak{R}}=\bigcup\nolimits_{j=1}^J \mathfrak{R}_j}$, and ${\bar{\varvec{A}}_f=[\bar{a}_{irf}] \in {\mathbb C}^{I \times R}}$ is created from the columns of the matrix $\varvec{A}_f$. For example, assuming ${\forall j: \mathfrak{R}_j=\{1, \ldots, \bar{R}\}}$, we have $R=J \bar{R}$, and ${\bar{\varvec{A}}_f=[\varvec{A}_f, \ldots, \varvec{A}_f] \in {\mathbb C}^{I \times R}}$ is the augmented mixing matrix [23] created from $\bar{R}$ matrices $\varvec{A}_f$. From (8) and (9), we have $s_{jft} \sim \mathcal{N}_c(0, \varvec{\Upsigma}_{ft}^{(s)})$ where ${\varvec{\Upsigma}_{ft}^{(s)}=\hbox{diag}\left (\left [\sum\nolimits_{r \in \mathfrak{R}_j} w_{fr} h_{rt} \right]_j \right)}$.

To estimate the parameters ${\mathcal{A}=[a_{ijf}] \in {\mathbb C}^{I \times J \times F}, \mathcal{C}=[c_{rft}] \in {\mathbb C}^{R \times F \times T}, \varvec{W} \in {\mathbb R}_+^{F \times R}, \varvec{H} \in {\mathbb R}_+^{R \times T}}$, and ${\varvec{\Upsigma}_n \in {\mathbb R}_+^{I \times I}}$, we formulate the following posterior:

10 $$ P({\mathcal{C}},\varvec{W},\varvec{H}|{\mathcal{X}},{\mathcal{A}},\varvec{\Upsigma}_n)= \frac{P({\mathcal{X}}|{\mathcal{C}}, {\mathcal{A}},\varvec{\Upsigma}_n) P({\mathcal{C}}|\varvec{W},\varvec{H}) P(\varvec{W}) P(\varvec{H})}{P({\mathcal{X}}|{\mathcal{A}}, \varvec{\Upsigma}_n)}, $$

from which we obtain

11 $$ \begin{aligned} \ln P({\mathcal{X}},{\mathcal{C}},\varvec{W},\varvec{H}|{\mathcal{A}},\varvec{\Upsigma}_n)&= \ln P({\mathcal{X}}|{\mathcal{C}},{\mathcal{A}},\varvec{\Upsigma}_n)+\ln P({\mathcal{C}}|\varvec{W},\varvec{H}) \\ &\quad+ \ln P(\varvec{W})+\ln P(\varvec{H}). \end{aligned} $$

From (3) and (9), we have the joint conditional PDF for $\mathcal{X}$:

12 $$ P({\mathcal{X}}|{\mathcal{C}},{\mathcal{A}},\varvec{\Upsigma}_n)=\prod_{i, f, t} {\mathcal{N}}_c \left (\sum_{r=1}^{R} \bar{a}_{irf} c_{rft}, \sigma^2_{i} \right)=\prod_{f, t} {\mathcal{N}}_c(\varvec{A}_f \varvec{s}_{ft}, \varvec{\Upsigma}_n). $$

Based on (12), the log-likelihood term in (11) can be expressed as

13 $$ \begin{aligned} \ln P({\mathcal{X}}|{\mathcal{C}},{\mathcal{A}},\varvec{\Upsigma}_n)&= - \sum_{f,t} (\varvec{x}_{ft} - \bar{\varvec{A}}_f \varvec{c}_{ft})^H \varvec{\Upsigma}_n^{-1}(\varvec{x}_{ft} - \bar{\varvec{A}}_f \varvec{c}_{ft}) - \sum_{f,t} \ln \det \varvec{\Upsigma}_n \\ &= - \sum_{f,t} (\varvec{x}_{ft} - \varvec{A}_f \varvec{s}_{ft})^H \varvec{\Upsigma}_n^{-1}(\varvec{x}_{ft} - \varvec{A}_f \varvec{s}_{ft}) \\ &\quad-\sum_{f,t} \ln \det \varvec{\Upsigma}_n+\hbox{const},\\ \end{aligned} $$

where $${\varvec{c}_{ft}=[c_{1ft}, \ldots, c_{Rft}]^T \in {\mathbb C}^R, \varvec{s}_{ft}=[s_{1ft}, \ldots, s_{Jft}]^T \in {\mathbb C}^J}$$, and $${\varvec{x}_{ft}=[x_{1ft}, \ldots, x_{Ift}]^T \in {\mathbb C}^I}$$.

The joint conditional PDF for $\mathcal{C}$ comes from the model (5):

14 $$ \begin{aligned} P({\mathcal{C}}|\varvec{W},\varvec{H})&= \prod_{r=1}^R \prod_{f=1}^F \prod_{t=1}^T {\mathcal{N}}_c \left (0, w_{fr} h_{rt} \right) \\ &= \prod_{r=1}^R \prod_{f=1}^F \prod_{t=1}^T |\pi w_{fr} h_{rt}|^{-1} \exp \left \{- \frac{|c_{rft}|^2 }{w_{fr} h_{rt}} \right \}. \end{aligned} $$

From (14), we have the log-likelihood functional for $\mathcal{C}:$

15 $$ \ln P({\mathcal{C}}|\varvec{W},\varvec{H})=- \sum_{r,f,t} \left (\ln(w_{fr}h_{rt})+\frac{|c_{rft}|^2}{w_{fr}h_{rt}} \right )+\hbox{const}. $$

The negative log-likelihood in (15) is the Itakura-Saito (IS) divergence [35], which is particularly useful for measuring the goodness of fit between spectrograms. The IS divergence is the special case of the β-divergence when $\beta \rightarrow -1$ [26].

The priors $P(\varvec{W})$ and $P(\varvec{H})$ in (10) can be determined in many ways. Févotte et al. [28] proposed the determination of priors using Markov chains and the inverse Gamma distribution. In our approach, we propose to model the priors with the Gibbs distribution, which is particularly useful for enforcing local smoothness in images.

## MRF Smoothing

Let us assume that prior information on the total smoothness of the estimated components ***W*** and ***H*** is modeled using the following Gibbs distributions:

16 $$ P(\varvec{W})=\frac{1}{Z_W} \exp \left \{-\alpha_W U(\varvec{W}) \right \}, \quad P(\varvec{H})=\frac{1}{Z_H} \exp \left \{-\alpha_H U(\varvec{H}) \right \} $$

where *Z*_*W*_ and *Z*_*H*_ are partition functions, α_*W*_ and α_*H*_ are regularization parameters, and *U*(***P***) is a total energy function, which measures the total roughness in ***P***. The function *U*(***P***) is often formulated with respect to the MRF model, which is commonly used in image reconstruction for modeling local smoothness.

The functions *U*(***W***) and *U*(***H***) can be determined for the matrices ***W*** and ***H*** in the following way:

17 $$ U(\varvec{W})= \sum_{f,r} \sum_{l \in S_f} \nu_{fl} \psi \left(w_{fr} - w_{lr}, \delta_W \right), $$

18 $$ U(\varvec{H})= \sum_{t,r} \sum_{l \in S_t} \nu_{tl} \psi \left(h_{rt} - h_{rl}, \delta_H \right). $$

In the first-order interactions (nearest neighborhood), we have *S*_*f*_ = {*f* − 1, *f* + 1} and the weighting factor ν_*fl*_ = 1, and *S*_*t*_ = {*t* − 1, *t* + 1} with ν_*tl*_ = 1. In the second-order interactions, *S*_*f*_ = {*f* − 2, *f* − 1, *f* + 1, *f* + 2} and *S*_*t*_ = {*t* − 2, *t* − 1, *t* + 1, *t* + 2}. The parameters δ_*W*_ and δ_*H*_ are scaling factors, while $\psi \left(\xi, \delta \right)$ is a potential function of ξ that can take different forms. The potential functions that can be applied to the EM-NMF algorithm are listed in Table 1.

**Table 1:** Potential functions

Author(s) (name)	Functions: ψ(ξ, δ)	Reference
(Gaussian)	(ξ/δ)^2^	
Besag (Laplacian)	$\left|\xi/\delta \right|$	[36]
Bouman and Sauer (GGMRF)	|ξ/δ|^*p*^	[37]
Geman and McClure	$\frac{16}{3 \sqrt{3}}\frac{(\xi/\delta)^2}{(1+(\xi/\delta)^2 )}$	[38]
Geman and Reynolds	$\frac{|\xi/\delta|}{1+|\xi/\delta|}$	[39]
Green	$\delta \ln [\cosh(\xi/\delta)]$	[34]
Hebert and Leahy	$\delta \ln [1+(\xi/\delta)^2]$	[40]

According to Lange [41], a robust potential function in the Gibbs prior should have the following properties: nonnegative, even, 0 at ξ = 0, strictly increasing for ξ > 0, unbounded, and convex with bounded first-derivative. Of the functions listed in Table 1, Green’s function satisfies all of these properties, and consequently, it was selected for use in the tests in [33]. Unfortunately, the application of Green’s function to both matrices ***W*** and ***H*** demands the determination of two hyperparameters δ_*W*_ and δ_*H*_, and two penalty parameters α_*W*_ and α_*H*_. Moreover, data-driven hyperparameter estimation usually involves an approximation of the partition functions $\varvec{Z}_W$ and $\varvec{Z}_H$, which is not easy in this task.

The Gaussian function $\psi \left(\xi, \delta \right)=\left(\xi/\delta \right)^2$, as shown in Table 1, does not have a bounded first-derivative, but its scaling parameter δ may be merged with a penalty parameter α. Consequently, only two parameters need to be determined. The MRF model with a Gaussian potential function is actually the SAR model [42–44], which is used widely in many scientific fields [44, 45] to represent the interactions among spatial data with Gaussian noise. Let ${\varvec{w}_r \in {\mathbb R}^F_+}$ be the *r*-th column of the matrix ***W***, and ${\underline {{\bf h}}_r \in {\mathbb R}^{1 \times T}_+}$ be the *r*-th row of the matrix ***H***. Random variables in the vectors $\varvec{w}_r$ and $\underline{{\bf h}}_r$ can be modeled using the following stochastic equations:

19 $$ \varvec{w}_r=\varvec{S}^{(W)} \varvec{w}_r+\epsilon, \quad \underline {{\bf h}}_r=\underline {{\bf h}}_r \varvec{S}^{(H)}+\epsilon, $$

where ${\varvec{S}^{(W)} \in {\mathbb R}^{F \times F}}$ and ${\varvec{S}^{(H)} \in {\mathbb R}^{T \times T}}$ are symmetric matrices of spatial dependencies between the random variables, $\epsilon \sim \mathcal{N}(0, \sigma^2 {\bf I})$ is an i.i.d. Gaussian noise, and **I** is an identity matrix with a corresponding size.

According to [45, 46], the spatial dependence matrices can be expressed as $\varvec{S}^{(W)}=\gamma \varvec{Z}^{(W)}$ and $\varvec{S}^{(H)}=\gamma \varvec{Z}^{(H)}$, where γ is a constant that ensures that the matrices $\varvec{C}^{(W)}={\bf I}_F - \varvec{S}^{(W)}$ and $\varvec{C}^{(H)}={\bf I}_T - \varvec{S}^{(H)}$ are positive-definite, while $\varvec{Z}^{(W)}=[z_{mf}^{(W)}]$ and $\varvec{Z}^{(H)}=[z_{tn}^{(H)}]$ are binary symmetric band matrices indicating the neighboring entries in $\varvec{w}_r$ and $\underline {{\bf h}}_r$, respectively. In the first-order interactions, we have *z*_1,2_^(*W*)^ = *z*_*F*,*F*-1_^(*W*)^ = *z*_*m*,*m*-1_^(*W*)^ = *z*_*m*,*m*+1_^(*W*)^ = 1 for $m \in \{2, \ldots, F-1 \}; z_{2,1}^{(H)}=z_{T-1,T}^{(H)}=z_{n-1,n}^{(H)}=z_{n+1,n}^{(H)} =1$ for $n \in \{2, \ldots, T-1 \}$, and *z*_*mf*_ = *z*_*tn*_ = 0 otherwise. In the *P*-order interactions, each entry *w*_*fr*_ and *h*_*rt*_ has the corresponding sets of neighbors: {*w*_*f*-ν,*r*_}, {*w*_*f*+ν,*r*_}, {*h*_*r*,*t*-ν_}, {*h*_*r*,*t*+ν_} with $\nu=1, \ldots, P$. As a consequence, $\varvec{Z}^{(W)}$ and $\varvec{Z}^{(H)}$ are symmetric band matrices with *P* sub-diagonals and *P* super-diagonals, the entries of which are equal to ones, but zeros otherwise. The matrices $\varvec{C}^{(W)}$ and $\varvec{C}^{(H)}$ are positive-definite, if γ < (2*P*)^−1^ for *P*-order interactions [45, 46]. We selected $\gamma=(2P)^{-1} - \tilde \epsilon$, where $\tilde \epsilon$ is a small constant, for example, $\tilde \epsilon=10^{-16}$.

In the SAR model, Gibbs priors (16) may be expressed as their joint multivariate Gaussian priors:

20 $$ P(\varvec{W})= \prod_{r=1}^R Z_W^{-1} \exp \left \{- \frac{\alpha_W}{2} ||\varvec{C}^{(W)} \varvec{w}_r||_2^2 \right \}, $$

21 $$ P(\varvec{H})= \prod_{r=1}^R Z_H^{-1} \exp \left \{- \frac{\alpha_H}{2} ||\underline {{\bf h}}_r \varvec{C}^{(H)}||_2^2 \right \} $$

where $Z_W=\left (\frac{2\pi}{\alpha_W} \right)^{F/2} \left (\prod\nolimits_{f=1}^F \lambda_f^2(\varvec{C}^{(W)}) \right)^{-1/2}$, and $\lambda_f(\varvec{C}^{(W)})$ is the *f*-th eigenvalue of the matrix $\varvec{C}^{(W)}$. Similarly, $Z_H=\left (\frac{2\pi}{\alpha_H} \right)^{T/2} \left (\prod_{t=1}^T \lambda_t^2(\varvec{C}^{(H)}) \right)^{-1/2}$. If $P=1: \lambda_f(\varvec{C}^{(W)}) \cong 1 - \cos \left (\frac{\pi f}{F} \right)$ and $\lambda_t(\varvec{C}^{(H)}) \cong 1 - \cos \left (\frac{\pi t}{T} \right)$, which simplifies the hyperparameter estimation.

## Algorithm

The EM algorithm [47] is applied to maximize $\ln P(\mathcal{X},\mathcal{C},\varvec{W},\varvec{H}|\mathcal{A},\varvec{\Upsigma}_n)$ in (11). To calculate the E-step, the log-likelihood functional (13) is transformed to the following form

22 $$ \begin{aligned} \ln P({\mathcal{X}}|{\mathcal{C}},{\mathcal{A}},\varvec{\Upsigma}_n)&= - T \sum_f \hbox{tr}\left \{\varvec{\Upsigma}_n^{-1} {\bf R}_f^{(xx)} \right \}+ T \sum_f \hbox{tr}\left \{\varvec{A}_f^H \varvec{\Upsigma}_n^{-1} {\bf R}_f^{(xs)} \right \} \\ &\quad + T \sum_f \hbox{tr}\left \{\varvec{\Upsigma}_n^{-1} \varvec{A}_f ({\bf R}_f^{(xs)})^H \right \} - T \sum_f \hbox{tr}\left \{\varvec{A}_f^H \varvec{\Upsigma}_n^{-1} \varvec{A}_f {\bf R}_f^{(ss)} \right \} \\ &\quad -\sum_{f,t} \ln \det \varvec{\Upsigma}_n, \end{aligned} $$

where the correlation matrices are given by ${\bf R}_f^{(xx)}=\frac{1}{T} \sum_t \varvec{x}_{ft} \varvec{x}_{ft}^H, {\bf R}_f^{(ss)}=\frac{1}{T} \sum_t \varvec{s}_{ft} \varvec{s}_{ft}^H$, and the cross-correlation ${\bf R}_f^{(xs)}=\frac{1}{T} \sum_t \varvec{x}_{ft} \varvec{s}_{ft}^H$.

Ozerov et al. [23] observed that the set {**R**_*f*_^(*xx*)^, **R**_*f*_^(*xs*)^, **R**_*f*_^(*ss*)^, |*c*_*rft*_|^2^ } provides sufficient statistics for the exponential family [47], so the sources $\varvec{s}_{ft}$ and the latent components $\varvec{c}_{ft}$ can be estimated by computing the conditional expectations of the natural statistics. According to [23], we have the following posterior estimates:

23 $$ \hat{\varvec{s}}_{ft}=\varvec{\Upsigma}_{ft}^{(s)} \varvec{A}_f^H (\varvec{A}_f \varvec{\Upsigma}_{ft}^{(s)} \varvec{A}_f^H+\varvec{\Upsigma}_n)^{-1} \varvec{x}_{ft}, $$

24 $$ \hat{\varvec{\Upsigma}}^{(s)}_{ft}=\varvec{\Upsigma}^{(s)}_{ft} - \varvec{\Upsigma}^{(s)}_{ft} \varvec{A}_f^H (\varvec{A}_f \varvec{\Upsigma}^{(s)}_{ft} \varvec{A}_f^H+\varvec{\Upsigma}_n)^{-1} \varvec{A}_f \varvec{\Upsigma}^{(s)}_{ft}. $$

Similarly, for the latent components, we have

25 $$ \hat{\varvec{c}}_{ft}=\varvec{\Upsigma}^{(c)}_{ft} \bar{\varvec{A}}_f^H (\bar{\varvec{A}}_f \varvec{\Upsigma}^{(c)}_{ft} \bar{\varvec{A}}_f^H+\varvec{\Upsigma}_n)^{-1} \varvec{x}_{ft}, $$

26 $$ \hat{\varvec{\Upsigma}}^{(c)}_{ft}=\varvec{\Upsigma}^{(c)}_{ft} - \varvec{\Upsigma}^{(c)}_{ft} \bar{\varvec{A}}_f^H (\bar{\varvec{A}}_f \varvec{\Upsigma}^{(c)}_{ft} \bar{\varvec{A}}_f^H+\varvec{\Upsigma}_n)^{-1} \bar{\varvec{A}}_f \varvec{\Upsigma}^{(c)}_{ft}. $$

The conditional expectations for the sufficient statistics are as follows:

27 $$ \hat{{\bf R}}_f^{(xx)}={\bf R}_f^{(xx)}, \quad \hat{{\bf R}}_f^{(xs)}=\frac{1}{T} \sum_t \varvec{x}_{ft} {\mathcal{E}}({\varvec{s}}_{ft}^H)=\frac{1}{T} \sum_t \varvec{x}_{ft} \hat{\varvec{s}}_{ft}^H, $$

28 $$ \hat{{\bf R}}_f^{(ss)}=\frac{1}{T} \sum_t {\mathcal{E}}(\varvec{s}_{ft}) {\mathcal{E}}({\varvec{s}}_{ft}^H)+\hat{\varvec{\Upsigma}}^{(s)}_{ft}=\frac{1}{T} \sum_t \hat{\varvec{s}}_{ft} \hat{\varvec{s}}_{ft}^H+\hat{\varvec{\Upsigma}}^{(s)}_{ft}, $$

29 $$ |c_{rft}|^2 \leftarrow {\mathcal{E}}(c_{rft}) {\mathcal{E}}(c_{rft}^H)+(\hat{\varvec{\Upsigma}}^{(c)}_{ft})_{rr}= |\hat{c}_{rft}|^2+(\hat{\varvec{\Upsigma}}^{(c)}_{ft})_{rr}. $$

Detailed derivations of the formulae (23)–(26) are presented in the ``Appendix''.

From the M-step, we have $\frac{\partial}{\partial \varvec{A}_f} \ln P(\mathcal{X},\mathcal{C},\varvec{W},\varvec{H}|\mathcal{A},\varvec{\Upsigma}_n)=2T(- \varvec{\Upsigma}_n^{-1} {\bf R}^{(xs)}_f+\varvec{\Upsigma}_n^{-1} \varvec{A}_f {\bf R}^{(ss)}_f)=0$, which gives $\varvec{A}_f=\hat{{\bf R}}^{(xs)}_f (\hat{{\bf R}}^{(ss)}_f)^{-1}$. From

$$ \frac{\partial}{\partial \varvec{\Upsigma}_n^{-1}} \ln P({\mathcal{X}},{\mathcal{C}},\varvec{W},\varvec{H}|{\mathcal{A}},\varvec{\Upsigma}_n)=0, $$

we have

$$ \varvec{\Upsigma}_n=\hbox{diag}\left \{{\bf R}^{(xx)}_f - \varvec{A}_f (\hat{{\bf R}}^{(xs)}_f)^H - \hat{{\bf R}}^{(xs)}_f \varvec{A}_f^H+\varvec{A}_f \hat{{\bf R}}^{(ss)}_f \varvec{A}_f^H \right \}. $$

From $\frac{\partial}{\partial w_{fr}} \ln P(\mathcal{X},\mathcal{C},\varvec{W},\varvec{H}|\mathcal{A},\varvec{\Upsigma}_n)=0$, we have

30 $$ w_{fr}=\frac{1}{T} \sum_{t=1}^T \frac{|c_{rft}|^2}{h_{rt}} - \alpha_W \nabla_{w_{fr}} U(\varvec{W}). $$

Similarly, from $\frac{\partial}{\partial h_{rt}} \ln P(\mathcal{X},\mathcal{C},\varvec{W},\varvec{H}|\mathcal{A},\varvec{\Upsigma}_n)=0$, we get

31 $$ h_{rt}=\frac{1}{F} \sum_{f=1}^F \frac{|c_{rft}|^2}{w_{fr}} - \alpha_H \nabla_{h_{rt}} U(\varvec{W}). $$

The terms $\nabla_{w_{fr}} U(\varvec{W})$ and $\nabla_{h_{rt}} U(\varvec{W})$ in (30) and (31) take the following forms with respect to the potential functions:

- *Gaussian* (SAR model):
  32 $$ \nabla_{w_{fr}} U(\varvec{W})= \left [(\varvec{C}^{(W)})^T \varvec{C}^{(W)} \varvec{W} \right]_{fr}, $$
  33 $$ \nabla_{h_{rt}} U(\varvec{H})= \left [\varvec{H} \varvec{C}^{(H)} (\varvec{C}^{(H)})^T \right]_{rt}, $$
- *GR function* (proposed by Green [34]):
  34 $$ \nabla_{w_{fr}} U(\varvec{W})= \sum_{l \in S_f} \nu_{fl} \tanh \left (\frac{w_{fr} - w_{lr}}{\delta_W} \right), $$
  35 $$ \nabla_{h_{rt}} U(\varvec{H})= \sum_{l \in S_t} \nu_{tl} \tanh \left (\frac{h_{rt} - h_{rl}}{\delta_H} \right). $$
- *HL function* (proposed by Hebert and Leahy [40]):
  36 $$ \nabla_{w_{fr}} U(\varvec{W})= \sum_{l \in S_f} \nu_{fl} \frac{2 \delta_W (w_{fr} - w_{lr})}{\delta^2_W+(w_{fr} - w_{lr})^2}, $$
  37 $$ \nabla_{h_{rt}} U(\varvec{H})= \sum_{l \in S_t} \nu_{tl} \frac{2 \delta_H (h_{rt} - h_{rl})}{\delta^2_H+(h_{rt} - h_{rl})^2}. $$

## Experiments

Experiments were conducted using selected sound recordings taken from the stereo audio source separation evaluation campaign (SiSEC)^1^ in 2007. This campaign aimed to evaluate the performance of source separation algorithms using stereo under-determined mixtures. We selected the benchmarks given in Table 2, which included speech recordings (three male voices—male3, and three female voices—female3), three nonpercussive music sources—nodrums, and three music sources that included drums—wdrums. The mixed signals were recordings that lasted 10 s, which were sampled at 16 kHz (the standard settings of recordings from the “Under-determined speech and music mixtures" datasets in the SiSEC2008). For each benchmark, the number of true sources was three (*J* = 3) but it only had two microphones (*I* = 2), that is, stereo recordings. Thus, for each case, we faced an under-determined BSS problem. All instantaneous mixtures were obtained using the same mixing matrix with positive coefficients. Synthetic convolutive mixtures were obtained for a meeting room with a 250 ms reverberation time using omnidirectional microphones with 1 m spacing.

**Table 2:** Benchmarks

Instantaneous	Convolutive
*male3*_*inst*_*mix*	*male3*_*synthconv*_*250ms*_*1m*_*mix*
*female3*_*inst*_*mix*	*female3*_*synthconv*_*250ms*_*1m*_*mix*
*nodrums*_*inst*_*mix*	*nodrums*_*synthconv*_*250ms*_*1m*_*mix*
*wdrums*_*inst*_*mix*	*wdrums*_*synthconv*_*250ms*_*1m*_*mix*

The spectrograms were obtained by a short-time fourier transform (STFT) using half-overlapping sine windows. To create the spectrograms and recover the time-domain signals from STFT coefficients, we used the corresponding stft_multi and istft_multi Matlab functions from the SiSEC2008 webpage^2^ [48]. For instantaneous and convolutive mixtures, the window lengths were set to 1,024 and 2,048 samples, respectively.

The EM-NMF algorithm was taken from Ozerov’s homepage^3^, while the MRF-EM-NMF algorithm was coded and extensively tested by Ochal [49].

The proposed algorithm is based on an alternating optimization scheme, which is intrinsically non-convex, and hence, its initialization plays an important role. An incorrect initialization may result in slow convergence and early stagnation at an unfavorable local minimum of the objective function. As done in many NMF algorithms, the factors ***W*** and ***H*** are initialized with uniformly distributed random numbers, whereas the entries in the matrix ***A*** are drawn from a zero-mean complex Gaussian distribution. After ***W*** and ***H*** have been initialized, the covariance matrices $\varvec{\Upsigma}_{ft}^{(s)}$ and $\varvec{\Upsigma}_{ft}^{(c)}$ given by (8) can be computed. A noise covariance matrix $\varvec{\Upsigma}_n$ is needed to update the E-step. Ozerov and Fevotte [23] tested several techniques for determining this matrix. The E-step in MRF-EM-NMF is identical to that in EM-NMF [23], and hence, all of these techniques can be used in this experiment. The initial matrix $\varvec{\Upsigma}_n$ was determined based on the empirical variance of the observed power spectrograms.

The MRF-EM-NMF and EM-NMF algorithms were initialized using the same random values (given as $\bar{R}$) and run for 1,500 iterations.

The choice of the parameters {α_*W*_, α_*H*_, γ_*W*_, γ_*H*_} used in the Gibbs distributions also affected the performance. The regularization parameters can be fixed or changed with iterations. Motivated by iterative thresholding strategies [26], we used the following strategies:

- Linear thresholding:
  $$ \alpha(k)=\alpha \frac{k}{k_{\rm max}}, $$
- Nonlinear thresholding:
  $$ \alpha(k)=\frac{\alpha}{2} \left (1+\tanh \left (\frac{k - \nu k_{\rm max}}{\tau k_{\rm max}} \right) \right), $$
- Fixed thresholding:
  $$ \alpha(k)=\left \{\begin{array}{cc}\alpha & \hbox{if }\quad k > k_1, \\ 0 & \hbox{otherwise} \end{array} \right. $$
  where *k* is the current iteration, *k*_max_ is the maximum number of iterations, $\tau \in (0,1)$ is the shape parameter, $\nu \in (0,1)$ is the shift parameter, *k*_1_ is the threshold, and α can be equal to α_*W*_ or α_*H*_. All of the above thresholding strategies aim to relax smoothing during the early iterations when the descent directions in the updates are sufficiently steep and to emphasize smoothing if noisy perturbations become significantly detrimental to the overall smoothness. These strategies are motivated by standard regularization rules that apply to ill-posed problems. We tested all of the thresholding strategies using instantaneous and convolutive mixtures, and we obtained the best performance with fixed thresholding using *k*_1_ = *k*_max_/2.

The parameters δ_*W*_ and δ_*H*_ in the MRF models can be estimated using standard marginalization procedures or by maximizing the Type II ML estimate for (10). However, these techniques have a huge computational cost for the nonlinear potential functions in the MRF models. For practical reasons, they are not very useful for the GR or HR functions.

In this study, we tested all of the benchmarks in Table 2 and the following potential functions: the first- and second-order Gaussian, GR, and HR. For the Gaussian functions, we tested all combinations of the regularization parameters α_*W*_ and α_*H*_ from the discrete set {0.001, 0.005, 0.01, 0.05, 0.1}. For GR and HL, the regularization parameters could take only two values, {0.001, 0.01}, although the parameters δ_*W*_ and δ_*H*_ were tested with the following values: {0.1, 1, 10}. The optimal values of the smoothing parameters are summarized in Table 3.

**Table 3:** Parameters of the MRF-EM-NMF algorithm for each test case shown in Fig. 1

Benchmark	Smoothing	Instantaneous mixture					Convolutive mixture				
		$\bar{R}$	α_*W*_	α_*H*_	δ_*W*_	δ_*H*_	$\bar{R}$	α_*W*_	α_*H*_	δ_*W*_	δ_*H*_
Male	GR	12	0.01	0.01	1	1	4	0.01	0.01	0.1	10
Male	HL	12	0.001	0.001	1	10	4	0.001	0.01	1	1
Male	1-Gaussian	12	0.001	0.01	–	–	4	0.05	0.05	–	–
Male	2-Gaussian	12	0.001	0.01	–	–	4	0.05	0.01	–	–
Female	GR	12	0.01	0.01	10	10	4	0.01	0.01	1	1
Female	HL	12	0.001	0.001	1	10	4	0.001	0.001	0.1	10
Female	1-Gaussian	12	0.001	0.001	–	–	4	0.1	0.001	–	–
Female	2-Gaussian	12	0.001	0.001	–	–	4	0.05	0.005	–	–
Nodrums	GR	4	0.01	0.01	10	1	4	0.01	0.01	10	0.1
Nodrums	HL	4	0.01	0.001	1	10	4	0.01	0.01	0.1	0.1
Nodrums	1-Gaussian	4	0.001	0.01	–	–	4	0.001	0.05	–	–
Nodrums	2-Gaussian	4	0.01	0.001	–	–	4	0.005	0.01	–	–
Wdrums	GR	4	0.01	0.01	1	10	4	0.01	0.01	1	1
Wdrums	HL	4	0.01	0.001	1	10	4	0.001	0.01	1	0.1
Wdrums	1-Gaussian	4	0.001	0.001	–	–	4	0.001	0.1	–	–
Wdrums	2-Gaussian	4	0.001	0.001	–	–	4	0.005	0.1	–	–

The notations "1-Gaussian" and "2-Gaussian" represent the first- and second-order Gaussian functions, respectively

**Fig. 1:**
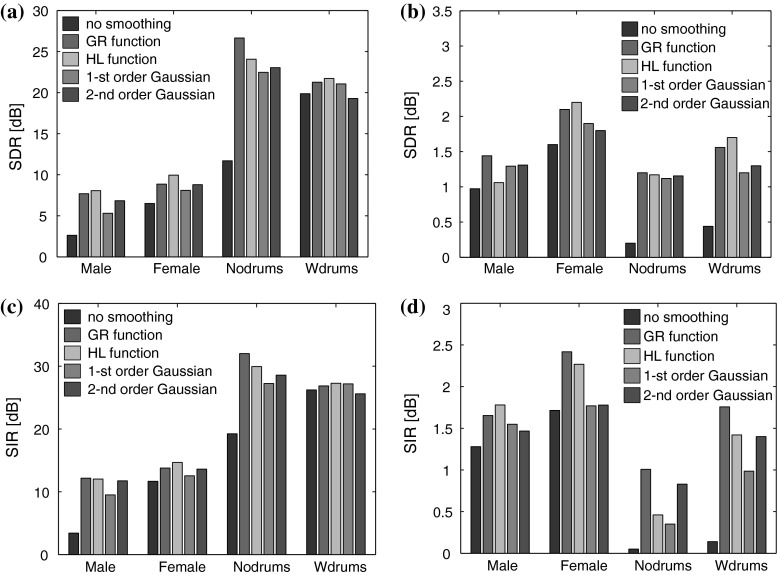
Source separation results obtained with the MRF-EM-NMF (first- and second-order Gaussian, GR, and HL functions) and EM-NMF (no smoothing) algorithms after 1,500 iterations: **a** mean SDR (dB) for instantaneous mixture, **b** mean SDR (dB) for convolutive mixture, **c** mean SIR (dB) for instantaneous mixture, **d** mean SIR (dB) for convolutive mixture. The smoothing parameters were tuned separately for each mixture in Table 2

**Fig. 2:**
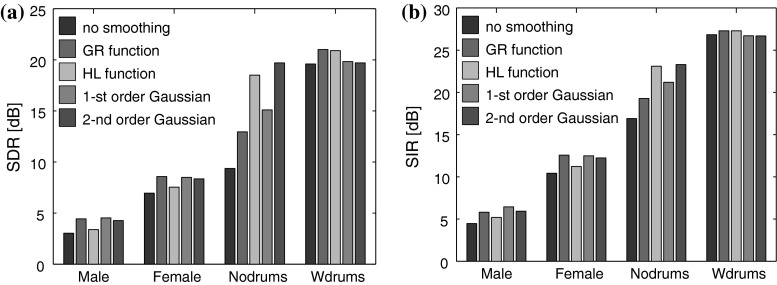
Source separation results obtained in the testing stage with the MRF-EM-NMF (first- and second-order Gaussian, GR, and HL functions) and EM-NMF (no smoothing) algorithm after 1,500 iterations: **a** mean SDR (dB), **b** mean SIR (dB). The smoothing parameters were determined during the training stage. 5 s excerpts were used in the training and testing stages

The separation results were evaluated in terms of the signal-to-distortion ratio (SDR) and the signal-to-interference ratio (SIR) [50]. Figure 1 shows the SDRs and SIRs averaged for the sources, which were estimated using the EM-NMF and MRF-EM-NMF with various smoothing functions based on instantaneous and convolutive mixing models. For each sample in Table 2 and each smoothing function, the smoothing parameters were tuned optimally for a given fixed initializer. This unsupervised learning approach evaluated the efficiency of the smoothing functions with respect to a given recording scenario. However, the smoothing parameters need to be determined with a supervised learning framework in practice. To test this option, each recording in Table 2 was divided into two 5 s excerpts during the training and testing stages. For each training excerpt, the smoothing parameters and initializer were selected to maximize the SDR performance. Testing was performed on the other excerpt with the same initializer. The results obtained during the testing stage with the instantaneous mixtures are shown in Fig. 2.

For comparison, Table 4 shows the average SDR results produced and the running time taken when using several state-of-the-art algorithms, which were applied to the mixtures in Table 2. The generalized Gaussian prior (GGP) algorithm [51] and the statistically sparse decomposition principle (SSDP) algorithms [52] were applied to the instantaneous mixtures. The convolutive mixtures were unmixed with the IPD [21], two versions of the FBWC-PA [17, 18] algorithm, and the Convolutive NMF [22]. Note that the last method in this list was based on supervised learning, whereas the others were unsupervised learning algorithms. In this case, the first 8 s excerpts of the 10 s source recordings were used for learning, while the remainder was used for testing.

**Table 4:** Mean SDR (dB) and running time (s) for sources estimated from the mixtures shown in Table 2

Benchmark	Mixture	Male	Female	Nodrums	Wdrums	Time
MRF-EM-NMF (HR)	inst	8.06	9.95	24.07	21.72	2487
MRF-EM-NMF (GR) [33]	inst	7.69	8.86	26.65	21.28	2498
EM-NMF [23]	inst	2.62	6.5	11.7	19.87	2456
GGP [51]	inst	8.4	8.57	13.9	10.3	5
SABM+SSDP [52]	inst	4.25	3.82	5.83	9.43	2
MRF-EM-NMF (HR)	conv	1.06	2.2	1.17	1.7	2760
MRF-EM-NMF (GR) [33]	conv	1.4	2.1	1.2	1.56	2762
EM-NMF [23]	conv	0.95	1.6	0.2	0.44	2720
IPD [21]	conv	1.53	1.43	2.2	−2.7	1200
FBWC-PA [17]	conv	−0.1	4.43	0.77	−2.53	40
Generalized FBWC-PA [18]	conv	5.95	7.45	1.2	−0.69	8
ConvNMF [22]	conv	−0.7	−0.47	3.85	8.13	347

The averaged elapsed time measured using Matlab 2008a for 1,500 iterations with $\bar{R}=12$, executed on a 64-bit Intel Quad Core CPU 3 GHz with 8 GB RAM was almost the same for the MRF-EM-NMF and EM-NMF algorithms (see Table 4).

The simulations demonstrate that MRF smoothing improved the source separation results in almost all test cases. The results confirmed that instantaneous mixtures were considerably easier to separate than convolutive ones. The MRF-EM-NMF algorithm delivered the best mean SDR performance of all the algorithms tested with instantaneous mixtures. The highest SDR values were produced with instantaneously mixed non-percussive music sources. This was justified by the smooth frequency and temporal structures of non-percussive music spectrograms. If the source spectrograms were not very smooth (as with the percussive audio recordings), MRF smoothing gave only a slight improvement (see Figs. 1, 2) in the first-order MRF interactions, and even a slight deterioration in the higher-order MRF interactions. According to Fig. 1, the HL function delivered the most promising SDR results, which were stable with a wide range of parameters. In each case with the instantaneous mixtures, the best results were produced with the same hyperparameter values, δ_*W*_ = 1 and δ_*H*_ = 10, and almost the same penalty parameter values, α_*W*_ and α_*H*_. The SAR model also improved the results compared with the standard EM-NMF algorithm. Moreover, the SAR model was tuned using only two penalty parameters, and the partition function of the associated Gibbs prior could be derived using a closed-form expression, which might be very useful for data-driven hyperparameter estimation.

The source separation results produced with the MRF-EM-NMF algorithm for convolutive and under-determined mixtures were better than those obtained with the EM-NMF algorithm. Unfortunately, the SDR values showed that these results were still a long way from being perfect, even after 1,500 iterations, and thus, further research is needed in this field. It is likely that some additional prior information could be imposed, especially on a mixing operator, which might increase the efficiency considerably.

It should be noted that the SDR performance with both mixtures could still be improved by refining the associated parameters, especially in the MRF models, and by using more efficient initializers.

## Conclusions

This study demonstrated that imposing MRF smoothing on the power spectrograms of audio sources estimated from under-determined unmixing problems may improve the quality of estimated audio sounds considerably. This was justified because any type of meaningful prior information improves the performance, especially with under-determined problems. This study addressed the application of MRF smoothing in the EM-NMF algorithm, but this type of smoothing could be applied to many other related BSS algorithms based on feature extraction from power spectrograms. Thus, the theoretical results presented in this paper may have broad practical applications. Clearly, further studies are needed to improve this technique for convolutive mixtures and to integrate regularization parameter estimation techniques in the main algorithm.

## Appendix

The conditional expectations of the natural statistics can be derived from the a posteriori distributions $P(\varvec{s}_{ft}|\varvec{x}_{ft})$ and $P(\varvec{c}_{ft}|\varvec{x}_{ft})$. Thus,

38 $$ \begin{aligned} P(\varvec{s}_{ft}|\varvec{x}_{ft})&= \frac{P(\varvec{x}_{ft}, \varvec{s}_{ft})}{P(\varvec{x}_{ft})} \\ &= \frac{(\pi^{I+J} \det \varvec{\Upsigma}_{ft})^{-1} \exp \left \{- \left [\begin{array}{c} \bar{\varvec{x}}_{ft} \\ \bar{\varvec{s}}_{ft} \end{array} \right]^H (\varvec{\Upsigma}_{ft})^{-1} \left [\begin{array}{c} \bar{\varvec{x}}_{ft} \\ \bar{\varvec{s}}_{ft} \end{array} \right] \right \}}{(\pi^{I} \det \varvec{\Upsigma}_{ft}^{(x)})^{-1} \exp \left \{- (\bar{\varvec{x}}_{ft})^H (\varvec{\Upsigma}_{ft}^{(x)})^{-1} \bar{\varvec{x}}_{ft} \right \}} \\ &= (\pi^{J} \det {\bf \Upgamma}_{ft})^{-1} \exp \left \{- {\bf \Uppsi}_{ft} \right \}, \end{aligned} $$

where $$\bar{\varvec{x}}_{ft}=\varvec{x}_{ft} - \mathcal{E}(\varvec{x}_{ft}), \bar{\varvec{s}}_{ft}=\varvec{s}_{ft} - \mathcal{E}(\varvec{s}_{ft}), {\bf \Upgamma}_{ft}=\varvec{\Upsigma}_{ft}^{(s)} - \varvec{\Upsigma}_{ft}^{(sx)} (\varvec{\Upsigma}_{ft}^{(x)})^{-1} \varvec{\Upsigma}_{ft}^{(xs)}$$,

$$ \varvec{\Upsigma}_{ft}=\left [\begin{array}{cc} \varvec{\Upsigma}_{ft}^{(x)} & \varvec{\Upsigma}_{ft}^{(xs)} \\ \varvec{\Upsigma}_{ft}^{(sx)} & \varvec{\Upsigma}_{ft}^{(s)} \end{array} \right], \quad {\bf \Uppsi}_{ft}=\left [\begin{array}{c} \bar{\varvec{x}}_{ft} \\ \bar{\varvec{s}}_{ft} \end{array} \right]^H (\varvec{\Upsigma}_{ft})^{-1} \left [\begin{array}{c} \bar{\varvec{x}}_{ft} \\ \bar{\varvec{s}}_{ft} \end{array} \right] - (\bar{\varvec{x}}_{ft})^H (\varvec{\Upsigma}_{ft}^{(x)})^{-1} \bar{\varvec{x}}_{ft}. $$

We can transform $\varvec{\Upsigma}_{ft}^{-1}$ in (38) into the following form:

$$ \varvec{\Upsigma}_{ft}^{-1}=\left [\begin{array}{cc} \left (\varvec{\Upsigma}_{ft}^{(x)} - \varvec{\Upsigma}_{ft}^{(xs)} (\varvec{\Upsigma}_{ft}^{(s)})^{-1} \varvec{\Upsigma}_{ft}^{(sx)} \right)^{-1} & - (\varvec{\Upsigma}_{ft}^{(x)})^{-1} \varvec{\Upsigma}_{ft}^{(xs)} {\bf \Upgamma}_{ft}^{-1} \\ - {\bf \Upgamma}_{ft}^{-1} \varvec{\Upsigma}_{ft}^{(sx)}(\varvec{\Upsigma}_{ft}^{(x)})^{-1} & {\bf \Upgamma}_{ft}^{-1} \end{array} \right]. $$

Using the Woodbury matrix identity, we have

$$ (\varvec{\Upsigma}_{ft}^{(x)} - \varvec{\Upsigma}_{ft}^{(xs)} (\varvec{\Upsigma}_{ft}^{(s)})^{-1} \varvec{\Upsigma}_{ft}^{(sx)})^{-1}=(\varvec{\Upsigma}_{ft}^{(x)})^{-1}+(\varvec{\Upsigma}_{ft}^{(x)})^{-1} \varvec{\Upsigma}_{ft}^{(xs)} {\bf \Upgamma}_{ft}^{-1} \varvec{\Upsigma}_{ft}^{(sx)} (\varvec{\Upsigma}_{ft}^{(x)})^{-1}, $$

and finally,

39 $$ \begin{aligned} {\bf \Uppsi}_{ft}&= \left (\bar{\varvec{s}}_{ft} - \varvec{\Upsigma}_{ft}^{(sx)} (\varvec{\Upsigma}_{ft}^{(x)})^{-1} \bar{\varvec{x}}_{ft} \right)^H {\bf \Upgamma}_{ft}^{-1} \left (\bar{\varvec{s}}_{ft} - \varvec{\Upsigma}_{ft}^{(sx)} (\varvec{\Upsigma}_{ft}^{(x)})^{-1} \bar{\varvec{x}}_{ft} \right) \\ &= \left ({\varvec{s}}_{ft} - \hat{\varvec{s}}_{ft} \right)^H (\hat{\varvec{\Upsigma}}^{(s)}_{ft})^{-1} \left ({\varvec{s}}_{ft} - \hat{\varvec{s}}_{ft} \right), \end{aligned} $$

where

40 $$ \hat{\varvec{s}}_{ft}={\mathcal{E}}(\varvec{s}_{ft})+\varvec{\Upsigma}_{ft}^{(sx)} (\varvec{\Upsigma}_{ft}^{(x)})^{-1} \left (\varvec{x}_{ft} - {\mathcal{E}}(\varvec{x}_{ft})\right), $$

41 $$ \hat{\varvec{\Upsigma}}^{(s)}_{ft}={\bf \Upgamma}_{ft}=\varvec{\Upsigma}_{ft}^{(s)} - \varvec{\Upsigma}_{ft}^{(sx)} (\varvec{\Upsigma}_{ft}^{(x)})^{-1} \varvec{\Upsigma}_{ft}^{(xs)}. $$

Thus, $P(\varvec{s}_{ft}|\varvec{x}_{ft})=\mathcal{N}_c(\varvec{s}_{ft}; \hat{\varvec{s}}_{ft}, \hat{\varvec{\Upsigma}}^{(s)}_{ft})$. From (5), it follows that $\mathcal{E}(\varvec{s}_{ft})=0$, so $\mathcal{E}(\varvec{x}_{ft})=0$. Since the zero-mean noise **n**_*ft*_ from (3) is not correlated with $\varvec{s}_{ft}$, we have

42 $$ \begin{aligned} \varvec{\Upsigma}_{ft}^{(xs)}&= {\mathcal{E}} \left ((\varvec{x}_{ft} - {\mathcal{E}}(\varvec{x}_{ft})) (\varvec{s}_{ft} - {\mathcal{E}}(\varvec{s}_{ft}))^H \right) \\ &= {\mathcal{E}} \left ((\varvec{A}_f \varvec{s}_{ft}+{\bf n}_{ft})\varvec{s}_{ft}^H \right)=\varvec{A}_f {\mathcal{E}}(\varvec{s}_{ft}\varvec{s}_{ft}^H)+{\mathcal{E}}({\bf n}_{ft}\varvec{s}_{ft}^H)=\varvec{A}_f \varvec{\Upsigma}_{ft}^{(s)}. \end{aligned} $$

Inserting (42) and $\varvec{\Upsigma}_{ft}^{(sx)}=(\varvec{\Upsigma}_{ft}^{(xs)})^H= \varvec{\Upsigma}_{ft}^{(s)} \varvec{A}_f^H$ into (40) and (41), we obtain the update rules (23) and (24), respectively.

Analyzing $P(\varvec{c}_{ft}|\varvec{x}_{ft})$, one can obtain $P(\varvec{c}_{ft}|\varvec{x}_{ft})=\mathcal{N}_c(\varvec{c}_{ft}; \hat{\varvec{c}}_{ft}, \hat{\varvec{\Upsigma}}^{(c)}_{ft})$, which yields the update rules (25) and (26).

## Footnotes

1
http://sisec.wiki.irisa.fr.

2
http://sisec2008.wiki.irisa.fr.

3
http://www.irisa.fr/metiss/ozerov/.
